# Phenylalanine-Based AMPA Receptor Antagonist as the Anticonvulsant Agent with Neuroprotective Activity—In Vitro and In Vivo Studies

**DOI:** 10.3390/molecules27030875

**Published:** 2022-01-27

**Authors:** Gniewomir Latacz, Kinga Sałat, Anna Furgała-Wojas, Adrian Martyniak, Agnieszka Olejarz-Maciej, Ewelina Honkisz-Orzechowska, Ewa Szymańska

**Affiliations:** 1 Department of Technology and Biotechnology of Drugs, Jagiellonian University Medical College, 9 Medyczna St., 30-688 Krakow, Poland; gniewomir.latacz@uj.edu.pl (G.L.); adrian.martyniak@uj.edu.pl (A.M.); agnieszka.olejarz@uj.edu.pl (A.O.-M.); ewelina.honkisz@uj.edu.pl (E.H.-O.); 2 Department of Pharmacodynamics, Faculty of Pharmacy, Jagiellonian University Medical College, 9 Medyczna St., 30-688 Krakow, Poland; kinga.salat@uj.edu.pl (K.S.); anna.furgala@student.uj.edu.pl (A.F.-W.)

**Keywords:** AMPA antagonist, phenylalanine, neuroprotection, antioxidant activity, FRAP, ORAC-FL, MAO-B inhibition, anticonvulsant activity

## Abstract

Trying to meet the multitarget-directed ligands strategy, a series of previously described aryl-substituted phenylalanine derivatives, reported as competitive antagonists of α-amino-3-hydroxy-5-methyl-4-isoxazolepropionic acid (AMPA) receptors, were screened in vitro for their free-radical scavenging and antioxidant capacity in two different assays: ferric reducing antioxidant power (FRAP) and oxygen radical absorbance capacity fluorescent (ORAC-FL) assays. The most active antioxidants **1** and **8** were further examined to evaluate their neuroprotective properties in vitro. In this study, compound **1** showed a significant neuroprotective effect against the neurotoxin 6-hydroxydopamine in neuroblastoma SH-SY5Y and IMR-32 cell lines. Both compounds also showed prevention from high levels of reactive oxygen species (ROS) in SH-SY5Y cells. Furthermore, the desired monoamine oxidase B (MAO-B) inhibition effect (IC_50_ = 278 ± 29 nM) for **1** was determined. No toxic effects up to 100 µM of **1** and **8** against neuroblastoma cells were observed. Furthermore, in vivo studies showed that compound **1** demonstrated significant anticonvulsant potential in 6-Hz test, but in neuropathic pain models its antiallodynic and antihyperalgesic properties were not observed. Concluding, the compound **1** seems to be of higher importance as a new phenylalanine-based lead candidate due to its confirmed promise in in vitro and in vivo anticonvulsant activity.

## 1. Introduction

One of the effective ways to prevent glutamate-induced neurotoxicity observed in many neurodegenerative conditions such as Alzheimer’s disease (AD), amyotrophic lateral sclerosis (ALS) and parkinsonism (PD) [1,2,3,4] is to target overactivation of ionotropic glutamate receptors (iGluRs) that mediate most excitatory synaptic transmission in the central nervous system (CNS). The family of iGluRs, especially the so-called non-N-methyl-D-aspartate (non-NMDA) receptors: α-amino-3-hydroxy-5-methyl-4-isoxazolepropionic acid (AMPA) and kainate (KA) receptors, have also been implicated in the pathogenesis of many other diseases such as neuropathic pain [3,5,6,7], depression [8,9], and the spread of seizure and neuronal damage associated with epilepsy [1,2,10,11,12,13,14]. Therefore, the rationale for the development of AMPA/KA receptor ligands as potential drugs for the treatment of these conditions has previously been suggested [12,14].

In recent years, a great impact has been put on competitive and non-competitive AMPA receptor antagonists with a broad spectrum of neuroprotective and anticonvulsant activity, a good example of which is perampanel, approved as the first-in-class AMPA receptor antagonist for the adjunctive treatment of partial-onset seizures in the United States and Europe [1,10,11,15]. However, the neurodegenerative diseases have been shown to have a multifactorial pathogenic mechanism, and drugs hitting a single target may be inadequate and inefficient for their treatment [16]. Recent studies have demonstrated the direct association of mitochondrial dysfunction and oxidative stress with AD, PD, ALS, as well as epileptogenesis or acquired chronic epilepsy [17,18]. Moreover, monoamine oxidase (MAO), which catalyzes deamination of molecules such as dopamine and serotonin in the presence of oxygen, increases the level of hydrogen peroxide, causing oxidative stress and death of neurons. Therefore, MAO inhibition is an important drug target in the treatment of PD and depression [19].

In this context, compounds showing AMPA antagonism combined with additional activities such as antioxidant effect, preventive action towards reactive oxygen species (ROS) generation, neuroprotection or MAO inhibition seem to have a high potential in the treatment of various psychiatric and neurological disorders, according to the modern multitarget-directed ligands (MTDLs) strategy [16]. Taking this into account, we decided to evaluate the antioxidant activity in vitro for the series of previously described aryl-substituted phenylalanine derivatives (**1**–**22**) with micromolar affinity and preference for AMPA receptors [20,21,22]. The most active antioxidants in this study — compounds **1** and **8** — have been further tested to evaluate their ability to prevent neuroblastoma SH-SY5Y and IMR-32 cell lines from the toxic activity of neurotoxin 6-hydroxydopamine (6-OHDA). Studies on MAO-B inhibition and ROS-scavenging properties were also carried out for compounds **1** and **8**.

Excitotoxicity is one of the primary mechanisms underlying cell loss in a variety of central and peripheral nervous system disorders. Among several signaling pathways controlling excitotoxicity, which are due to the excessive release of glutamate from axon terminals or overactivation of NMDA receptors, Ca^2+^ influx-triggered excitotoxicity through AMPA receptors has been demonstrated in multiple disease models. These AMPA receptor-dependent phenomena are regarded as significant contributors to both acute brain injuries and chronic neurological disorders, such as epilepsy, neurodegenerative disorders (Huntington’s disease, PD, AD, ALS), chronic pain, and many others [23]. Since the in vivo part of the present research was the first-in-animal study and also considering that in vitro neuroprotective properties of compound **1** were assessed, we focused on in vivo assays to evaluate the compound **1** in animal models and tests that reflect disorders for which AMPA receptor antagonists seem to be particularly promising drug candidates, namely epilepsy [10,24] and pain [25,26,27].

Therefore, the in vivo part of this present research aimed to establish the ability of compound **1**, the most active antioxidant and, at the same time, the most potent AMPA receptor antagonist among the compounds tested in the present study**,** to prevent seizure episodes. To assess the effect of compound **1** on seizure threshold in mice, we used models of electrically induced seizures (i.e., maximal electroshock (MES) and 6-Hz tests) and chemically induced seizures (pentylenetetrazole (PTZ) test). To some degree, the mechanisms underlying epilepsy and neuropathic pain are similar, and therefore many antiepileptic drugs (AEDs) are used in some non-epileptic conditions, including neuropathic pain of various origin.

Moreover, considering a high rate of pharmacoresistance of both diseases, the number of drugs effectively alleviating seizures and pain symptoms is not sufficient and there is still a strong medical demand to search for novel drug candidates for these two neurological conditions. Thus, a model of neuropathic pain reflecting human diabetic and chemotherapy-induced neuropathic pain was additionally involved to assess whether compound **1** could attenuate pain hypersensitivity in neuropathic mice.

Additionally, selected physicochemical and pharmacokinetic parameters for compounds **1** and **8** were predicted in silico using the SwissADME website [28] and the results were compared with the descriptors calculated for the known phenylalanine-based anticonvulsant agent—levodopa ((*S*)-3,4-dihydroxyphenylalanine), used in the clinical treatment of Parkinson’s disease (Tables S1 and S2, Supplementary Materials). As it could be expected, none of these amino acids crosses the blood-brain barrier passively; however, as it was reported for levodopa drug that the neutral amino acids can be transported across the blood-brain barrier via the large neutral amino acid transporter type 1 [29,30,31].

## 2. Results and Discussion

The series of synthetic, phenylalanine-based α-amino acids acting as competitive AMPA/KA receptor antagonists was previously described [21,22,32,33]. Among them, the group of aryl-substituted phenylalanines seems to be the most interesting. Development of a structure-activity relationship for this series allowed us to discover that substitution with a hydroxy group at the distal aromatic moiety (especially at 3′-position of the distal phenyl ring) had a large impact on both affinity and selectivity of binding at AMPA receptors over KA and NMDA receptors. The combination of 3′-OH substitution with 5-NO_2_-6-Cl pattern at the phenylalanine ring resulted in the most potent AMPA ligands in the entire series: **1**, **2** and **18** (Table 1), with affinity in range of 0.92–3.1 μM and high selectivity over NMDA and KA receptors [22].

### 2.1. In Vitro Assays

#### 2.1.1. Antioxidant Activity

The racemic hydroxyaryl-substituted phenylalanines listed in Table 1 were screened for their antioxidant properties using two different methods: colorimetric-based ferric reducing antioxidant power (FRAP) assay [34] and oxygen radical absorbance capacity fluorescent (ORAC-FL) assay [35]. The results were compared with the antioxidant effect of the references: ascorbic acid (AA) and Trolox (TX) in FRAP and ORAC-FL, respectively. The obtained results, expressed as % of ascorbic acid activity (%AAA) or Trolox equivalent (TE), are presented in Table 1 in order of decreasing FRAP activity and compared with their affinity for native AMPA receptors.

As shown in Table 1, the antioxidant properties of phenylalanines established in the FRAP assay appear to be partially correlated with their AMPA receptor affinity. Thus, the tested phenylalanines can be roughly divided into three groups: (1) the most active compounds **1**–**4** with antioxidant activity higher than the reference AA and AMPA receptor affinity within the range of 0.92–12 μM; (2) compounds **5**–**14** with medium FRAP activity 20–99% of AA and AMPA receptor affinity *K*_i_ = 8–20 μM—with the exception of **7** and **8** (both compounds are 4′-hydroxyaryl substituted phenylalanines, less potent at AMPA receptors than their 3′-OH counterparts); (3) compounds **15**–**22**, showing antioxidant activity lower than 20% of AA and very weak binding at AMPA receptors (except for compounds **18** and **19** with higher AMPA receptor affinity). The highest FRAP activity was found for the two most potent AMPA receptor ligands within the entire series of phenylalanines tested, **1** and **2**, which contained the 5-NO_2–_6-Cl pattern at the phenylalanine ring and either 2′,5′-diOH-phenyl or 5′-OH-3-pyridyl as a distal aromatic moiety.

In the ORAC-FL assay, all examined compounds showed higher antioxidant activity than the reference TX, which is not surprising considering the presence of phenolic hydroxy groups that are known to enhance free-radical-scavenging properties of compounds [36,37,38]. However, the results observed in the ORAC-FL test do not follow the order of the antioxidant effect seen in the FRAP assay (Table 1). The most active in the FRAP test phenylalanines **1**–**4** demonstrate only medium activity in the ORAC-FL assay (TE in range of 3.15–4.61), while compounds **5**–**14** with medium FRAP activity show TX equivalent that varies from one of the lowest values (TE = 1.43 for **10**) to the highest values within the entire series (TE = 7.56 and 6.09 for the compounds **8** and **6**, respectively). The antioxidant effect of hydroxyaryl phenylalanines in this test appears to be enhanced the most by the presence of amino or a second hydroxy group at the specific 6-position of the phenylalanine ring—four out of five most active compounds with TE > 4.5 contain in the structure this type of substitution pattern (**8** > **6** > **14** > **9** > **3**). Surprisingly, compounds with two hydroxy groups located at the same distal phenyl ring (**1**, **4**, **11**) are less active and show TE in range of 3.46–4.18. On the other hand, in the FRAP test, the increase in antioxidant activity is observed for the compounds with multiple phenolic hydroxy groups, but their individual positions in the structure seem to have no significant meaning (**1**, **3**, **4**).

The third group of compounds, **15**–**22**, was found to have the weakest antioxidant capacity according to both assays.

The lack of convergence in the antioxidant activity results seen in the FRAP and ORAC-FL is probably related to principles of both methods used. The ORAC-FL method measures the prevention of free radicals generated by 2,2′-azobis-2-amidinopropane dihydrochloride (AAPH) at low concentrations of tested compounds (< 10 µM) [35], while in the FRAP method the compound’s ability to reduce ferric to ferrous ions at low pH is determined at the concentration of 1 mM [34].

Based on the results mentioned above, we selected for the next, cell-based neuroprotective studies the best antioxidant from each method: compound **1** from the FRAP test with 748.5% AAA and compound **8** from ORAC-FL with calculated TE = 7.56 (Table 1). It must be noted that compound **8** showed in the FRAP method only %AAA = 48.19 and lack of detectable AMPA receptor affinity. However, due to its high antioxidant effect in the ORAC-FL assay we decided to proceed this compound further.

**Table 1:**
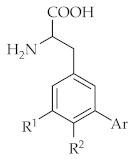
The structures of racemic phenylalanine derivatives **1**–**22** and their antioxidant properties compared to α-amino-3-hydroxy-5-methyl-4-isoxazolepropionic acid (AMPA) receptor affinity.

Cmpd.	R ^1^	R ^2^	Ar	FRAP (%AAA) ^1^	ORAC (TE) ^2^	[^3^H]AMPA *K_i_* (μM) ^3^
**1**	NO_2_	Cl	2′,5′-diOH Ph	748.49	3.46	0.92
**2**	NO_2_	Cl	5′-OH-3-pyridyl	691.21	3.15	2.0
**3**	Cl	OH	3′-OH Ph	311.56	4.61	7.9
**4**	Cl	Cl	3′,4′-diOH Ph	177.23	4.02	12 ^4^
**5**	NO_2_	H	4′-OH Ph	96.32	4.32	21
**6**	H	NH_2_	3′-OH Ph	67.35	6.09	9.1
**7**	Cl	H	4′-OH Ph	49.39	3.29	46
**8**	Cl	NH_2_	4′-OH Ph	48.19	7.56	>100
**9**	NO_2_	H	3′-OH Ph	43.43	4.67	8.2
**10**	H	NO_2_	3′-OH Ph	32.04	1.43	21
**11**	Cl	Cl	3′,5′-diOH Ph	28.90	4.18	16 ^4^
**12**	NO_2_	NH_2_	3′-OH Ph	24.93	4.27	14
**13**	H	Cl	3′-OH Ph	24.08	2.86	9.2
**14**	Cl	NH_2_	3′-OH Ph	23.80	4.75	10
**15**	Cl	Cl	4′-OH Ph	13.70	1.45	33 ^4^
**16**	Cl	Cl	2′-OH Ph	10.32	1.54	85 ^4^
**17**	H	Cl	4′-OH Ph	9.13	1.96	65
**18**	NO_2_	Cl	3′-OH Ph	6.29	1.34	3.1
**19**	H	H	3′-OH Ph	4.94	1.96	9.9 ^4^
**20**	H	NO_2_	4′-OH Ph	4.41	1.62	64
**21**	NO_2_	Cl	4′-OH Ph	2.76	2.56	23
**22**	Cl	OCH_3_	3′-OH Ph	2.76	1.38	>100

^1^ Ascorbic acid (AA) and compounds **1**–**22** were examined at 1 mM concentration; ^2^ The Trolox equivalent (TE) was calculated for results obtained at 5 µM concentration of compounds **1**–**22** and Trolox (TX); ^3^ Binding pharmacology at native AMPA receptors in rat brain membranes, the data are given as mean of three separate experiments [20,21,22]; ^4^ IC_50_ values.

#### 2.1.2. Neuroprotection

Neuroprotective assays were next performed in vitro using two neuroblastoma cell lines: dopaminergic, related to PD, SH-SY5Y cell line [39], and IMR-32 cell line, which expresses most of the proteins of cholinergic neurons and which was described as an in vitro model to study AD [40]. The cells were injured by the neurotoxin 6-hydroxydopamine (6-OHDA), commonly used in experimental animal models of PD [41,42]. According to the literature, 6-OHDA-induced apoptosis may be mediated by several effects, (i) high level of ROS derived from 6-OHDA autooxidation; (ii) high level of ROS obtained in the reaction catalyzed by MAO-A; (iii) the direct effect of 6-OHDA on the mitochondrial respiration [43,44].

**Figure 1:**
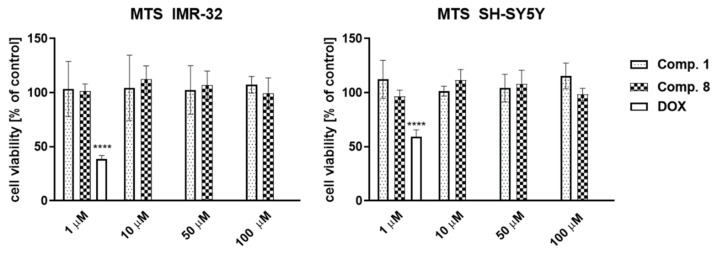
Viability of IMR-32 (left) and SH-SY5Y (right) neuroblastoma cells’ after 24 h of incubation with compounds **1**, **8** and the reference cytostatic drug doxorubicin (DOX, 1 µM). Statistical significance was evaluated by one-way ANOVA, followed by Bonferroni’s comparison (∗∗∗∗ *p* < 0.001).

The potential toxicity of the most potent antioxidants selected, namely compounds **1** and **8,** against both cell lines was examined first in a standard colorimetric cell proliferation assay using 3-(4,5-dimethylthiazol-2-yl)-5-(3-carboxymethoxyphenyl)-2-(4-sulfophenyl)-2H-tetrazolium salt (MTS reagent) in order to select the nontoxic concentrations for the neuroprotection assays. Figure 1 shows that after 24 h of cell incubation with compounds **1** and **8** in the concentration range 1–100 µM, no influence on cell viability was noted. Thus, for further neuroprotection studies 50 µM concentration of both compounds was selected.

**Figure 2:**
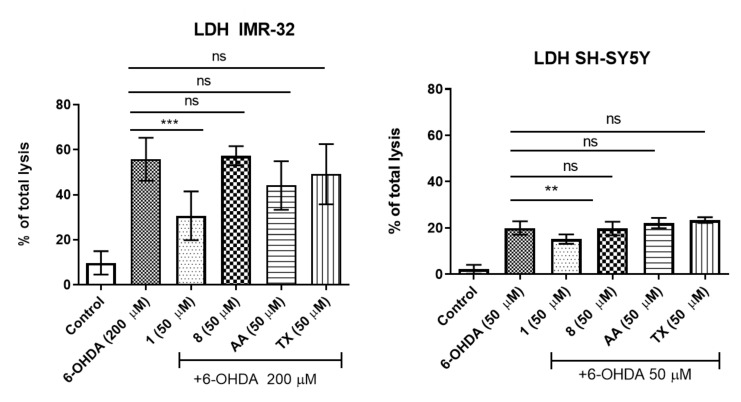
Neuroprotection studies of compounds **1** and **8** (50 µM) against 6-OHDA-induced LDH release from necrotic cells. Ascorbic acid (AA, 50 µM) and Trolox (TX, 50 µM) were used as the reference antioxidants. Statistical significance was evaluated by one-way ANOVA, followed by Bonferroni’s comparison (ns—not significant, ∗∗ *p* < 0.01, ∗∗∗ *p* < 0.001 compared with the positive control: 200 µM or 50 µM of 6-OHDA).

Our previous work reported the following concentrations of 6-OHDA suitable for investigation of compounds neuroprotection: 50 µM for SH-SY5Y and 200 µM for IMR-32 cell line, respectively [36]. The protective effects against the toxic activity of 6-OHDA neurotoxin in neuroblastoma cell lines were assessed next by the measurement of lactate dehydrogenase (LDH) release after 24 h of 6-OHDA incubation with or without presence of **1** and **8** (Figure 2). IMR-32 and SH-SY5Y cell lines were first pretreated with the phenylalanine derivatives for 1 h and then incubated together with 6-OHDA. The examined phenylalanine derivative **1** showed a statistically significant decrease of 6-OHDA-induced release of LDH in both cell lines; however, the observed neuroprotection of **1** was more effective in the case of IMR-32 cells. Interestingly, no statistically significant neuroprotective effect of compound **8** as well as reference antioxidants AA and TX (50 µM) was determined (Figure 2). The compounds were also tested in SH-SY5Y cells with use of higher concentration of 6-OHDA (100 µM); however, in this case the neuroprotective effect was not observed either for **1**, **8** or the reference antioxidants (Figure S1 in Supporting Materials).

#### 2.1.3. ROS Assay

**Figure 3:**
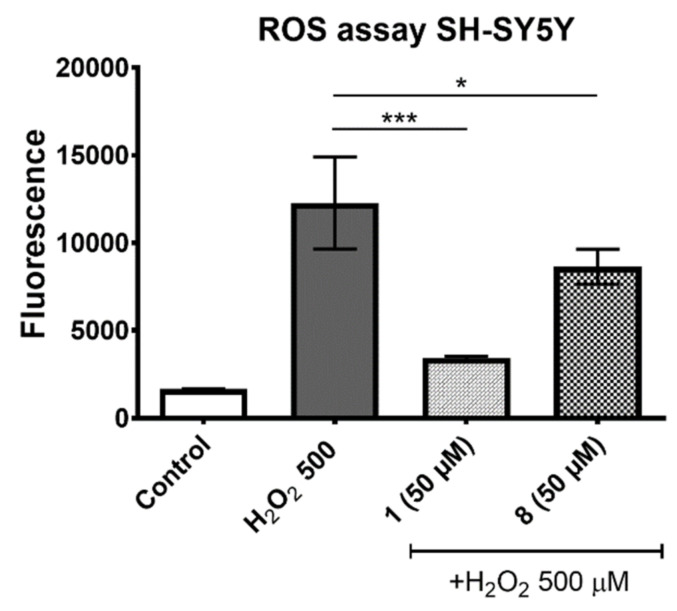
The protective effect of compounds **1** and **8** (50 µM) against H_2_O_2_-induced increase of intracellular ROS level. Statistical significance was evaluated by one-way ANOVA, followed by Bonferroni’s comparison (∗ *p* < 0.05, ∗∗∗ *p* < 0.001 compared with the positive control—cells treated with 500 µM H_2_O_2_).

The ROS and MAO-B assays were performed to understand the potential mechanism of compound’s **1** neuroprotection. In the ROS assay the protective effect against H_2_O_2_-induced ROS accumulation in SH-SY5Y cells was investigated. The used 2′,7′–dichlorofluorescein diacetate (DCFH-DA) was deacetylated in cells first and converted next quantitatively in the presence of ROS into the highly fluorescent 2′,7′-dichlorofluorescein (DCF). In Figure 3 the measured level of fluorescence in SH-SY5Y cells is shown after 3 h of H_2_O_2_ (500 µM) incubation with or without the presence of the selected phenylalanine derived antioxidants. The statistically significant decrease in the intracellular ROS level was observed for **1** and **8**, respectively. It should be emphasized that the better effect was determined for compound **1**, with the highest antioxidant action determined in the FRAP assay (Figure 3).

#### 2.1.4. MAO-B Inhibition

**Figure 4:**
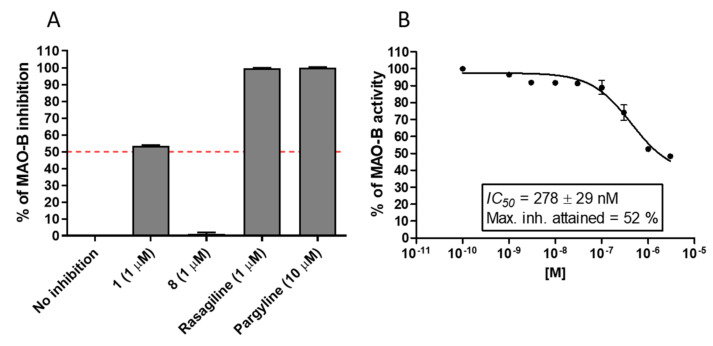
(**A**) Inhibition of MAO-B – screening of tested compounds and the reference inhibitors (rasagiline IC_50_ = 25 nM, pargyline IC_50_ = 652 nM); (**B**) Dose-dependent curve of compound **1**.

To determine the inhibitory potential of the investigated phenylalanine derivatives **1** and **8** towards MAO-B, the Amplex Red^®^ Monoamine Oxidase kit [45] was used according to the previously described protocol [46]. The compounds were first screened at 1 µM concentration, and compared to the MAO-B reference inhibitors, rasagiline and pargyline (Figure 4A). No inhibitory activity was determined for compound **8**, while compound **1** showed more than 50% of full inhibition (represented by pargyline 10 µM) and was selected for the experiment in an extended range of concentrations to determine IC_50_ value. The dose-dependent curve of **1** allowed for calculation of IC_50_ = 278 ± 29 nM (Figure 4B). However, compound **1** did not show full inhibition of MAO-B, and the maximum inhibition attained was 52%.

### 2.2. In Vivo Assays

Neuroprotective and antioxidant properties of the compound **1** shown in the in vitro part of the present research, combined with its previously revealed effect on AMPA receptors can be at least in part framed in the context of several psychiatric and neurological diseases, such as for example AD, PD, epilepsy and chronic pain. Therefore, the results obtained in vitro were the basis for the transfer of the compound **1** for further in vivo evaluation. In this first-in-animal set of experiments we focused on selected assays that could reveal activities strongly related to neuroprotection combined with AMPA receptor antagonism.

Compound **1** was therefore selected for this in vivo research in animal models of seizures and chronic pain. Neuroinflammation with activation of microglia and increased production of pro-inflammatory cytokines in the brain play a key role in epileptic seizures, and the oxidative stress in brain tissues has been implicated as one of the main causes of epilepsy. Oxidative stress in the hippocampus has been associated with temporal lobe epilepsy, a common form of epilepsy in humans [47], and it has also been demonstrated to be one of the factors involved in diabetic [48,49] and chemotherapy-induced [50] neuropathic pain. Importantly, epilepsy and neuropathic pain episodes are regarded as relatively frequent comorbidities in patients with PD and AD [51,52,53,54].

#### 2.2.1. Anticonvulsant Activity in Seizure Models

MES Test

In MES, test **1** was not effective. At the dose of 100 mg/kg it did not protect mice from seizure episodes. In addition, compared to the control group the compound **1** was not able to reduce mortality rate in this assay (Table 2).

PTZ Seizure Test

**Figure 5:**
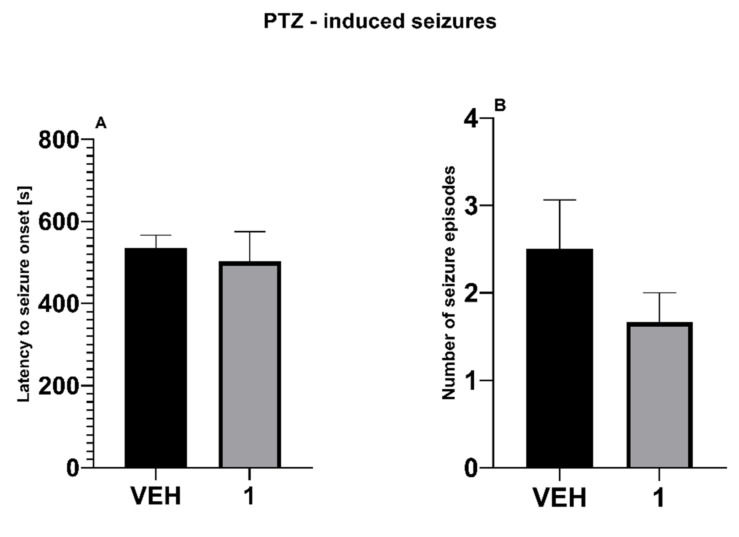
Influence of the test compound **1** at the dose of 100 mg/kg on latency to first clonus (**A**) and the number of seizure episodes (**B**) in PTZ-induced seizure model. Statistical analysis: Student’s *t*-test test. Significance vs. vehicle-treated mice: *p* > 0.05.

In PTZ test, the test compound was not able to prolong latency to the first clonus in PTZ-treated mice (Figure 5A). In addition, the number of seizure episodes remained unaffected (Figure 5B).

6-Hz Test

In the 6-Hz test, the anticonvulsant activity of compound **1** was observed at the dose of 100 mg/kg was (Table 3).

#### 2.2.2. Antiallodynic and Antihyperalgesic Activity in Neuropathic Pain Models

##### Diabetic Neuropathic Pain Model

Effect on Tactile Allodynia—Von Frey Test

**Figure 6:**
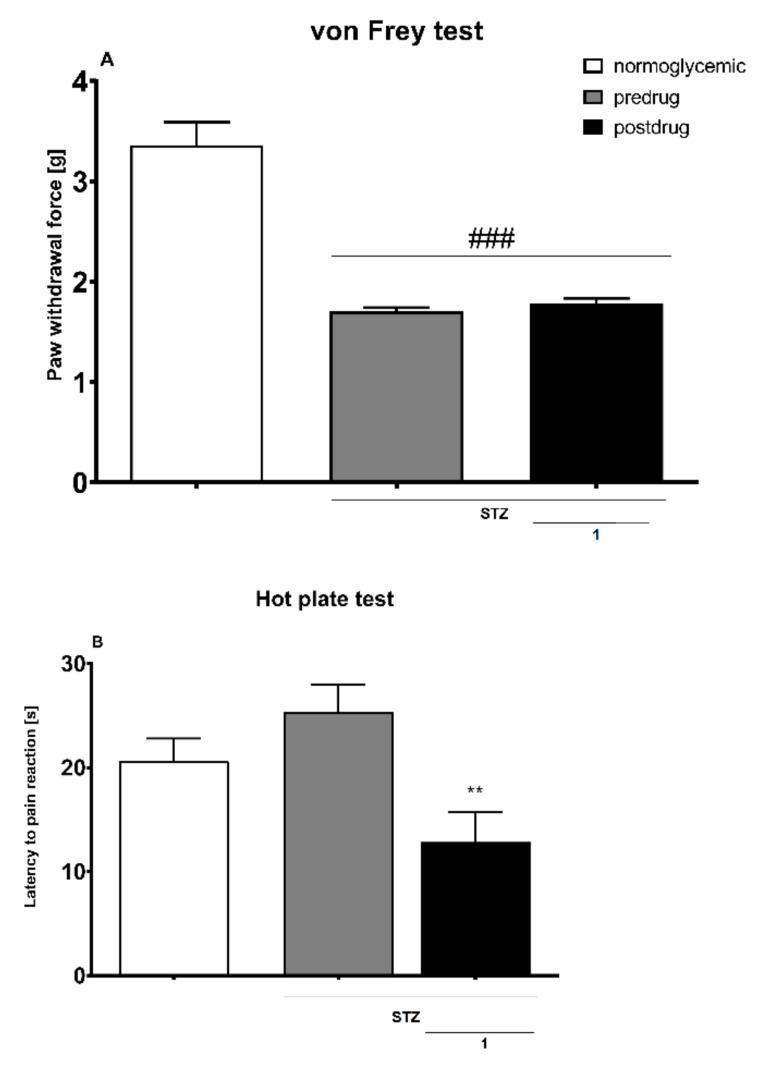
Assessment of antiallodynic activity of the compound **1** (30 mg/kg i.p.) in the diabetic neuropathic pain model measured using the von Frey test (**A**), and antihyperalgesic effect of this compound assessed in diabetic mice and measured using the hot plate test (**B**). Results are shown as the mean (± SEM) force applied to elicit paw withdrawal (**A**), or the mean (± SEM) latency to pain reaction (**B**). Statistical analysis: repeated measures ANOVA, followed by Tukey’s post hoc comparison. Significance vs. normoglycemic control: ### *p* < 0.001, and vs. pre-drug value in the individual group: ** *p* < 0.01. In the vehicle-treated normoglycemic mice measurements of pain sensitivity threshold were taken in the same manner as in the STZ group, but vehicle-treated mice were not treated with STZ.

In the von Frey test, one-way ANOVA revealed a significant effect of treatment (F[2,24] = 56.33, *p* < 0.0001). Streptozotocin (STZ) induced a significant (*p* < 0.0001) decrease of mechanical nociceptive threshold in mice. The compound **1** (30 mg/kg) was not able to attenuate tactile allodynia in diabetic neuropathic mice (Figure 6A).

Effect on Heat Hyperalgesia—Hot Plate Test

In the hot plate test, one-way ANOVA revealed a significant effect of treatment (F[2,24] = 6218, *p* < 0.01). Compared to normoglycemic mice, the latencies to pain reaction in STZ-treated mice did not differ significantly. In STZ-treated mice the compound **1** (30 mg/kg) significantly (*p* < 0.01 vs. pre-drug latency) reduced latency to pain reaction in the hot plate test, and therefore its antihyperalgesic activity was not observed (Figure 6B).

##### Oxaliplatin-Induced Neuropathic Pain Model

Effect on Tactile Allodynia—Von Frey Test

**Figure 7:**
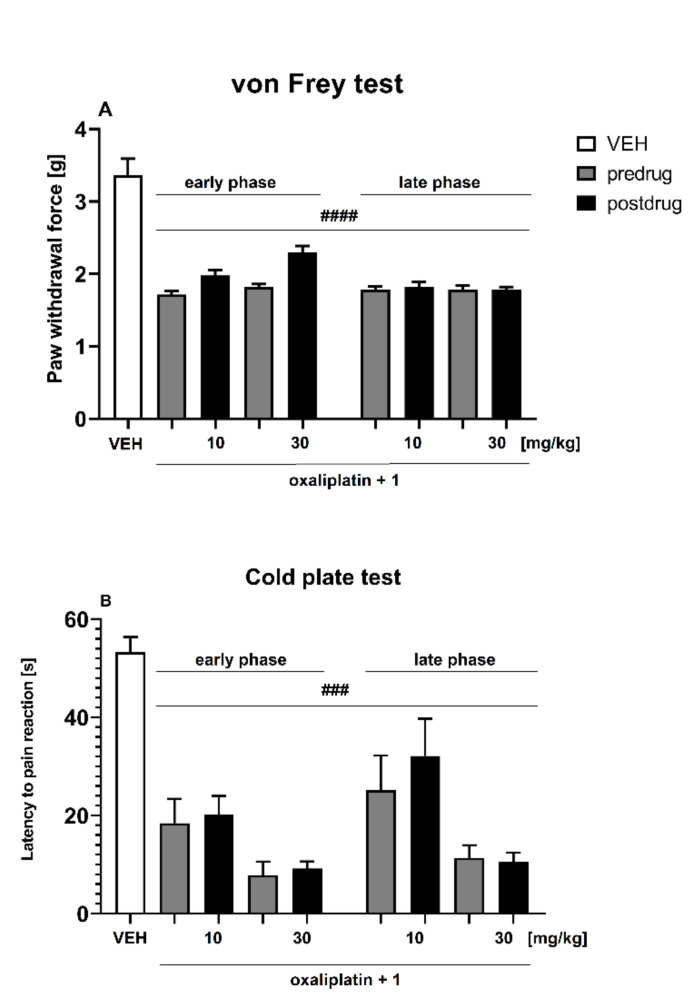
Effect of **1** (doses: 10 and 30 mg/kg i.p.) on pain threshold in oxaliplatin-induced neuropathic pain model measured using the von Frey test (**A**) and the cold plate test (**B**) in the early phase (3 h after oxaliplatin injection) and in the late phase (7 days after oxaliplatin injection) of neuropathy. Results are shown as the mean (±SEM) force applied to elicit paw withdrawal (**A**), or the mean (±SEM) latency to pain reaction (**B**). Statistical analysis: repeated measures ANOVA, followed by Tukey’s *post hoc* comparison. Significance vs. non-neuropathic, vehicle-treated mice (VEH): ### *p* < 0.001, #### *p* < 0.0001. In the VEH group, measurements of pain sensitivity threshold were taken in the same manner and at the same time points as in oxaliplatin-treated groups but vehicle-treated mice were not treated with oxaliplatin.

In the von Frey test, an overall effect of treatment was observed (F[2,130] = 82.65, *p* < 0.0001). Time also affected the results significantly (F[4,130] = 34.50, *p* < 0.0001), and drug × time interaction was significant (F[8,130] = 5.976, *p* < 0.0001). *Post hoc* analysis revealed that in mice oxaliplatin induced a statistically significant (*p* < 0.0001) reduction of mechanical nociceptive threshold both in the early and in the late phase of neuropathy. Compound **1** at doses of 10 and 30 mg/kg was not effective in this assay (Figure 7A).

Effect on Cold Hyperalgesia—Cold Plate Test

In the cold plate test, an overall effect of treatment was observed (F[2,105] = 102.1, *p* < 0.0001). Time also affected the results significantly (F[4,105] = 22.53, *p* < 0.0001) and drug × time interaction was significant (F[8,105] = 5.724, *p* < 0.0001). *Post hoc* analysis revealed that in mice oxaliplatin induced a statistically significant (*p* < 0.001) reduction of cold nociceptive threshold both in the early and in the late phases of neuropathy. Compound **1** at doses of 10 and 30 mg/kg was unable to attenuate cold hyperalgesia in this assay (Figure 7B).

## 3. Conclusions

In summary, the data obtained in vitro identified all examined phenylalanines **1**–**22** as antioxidants equal to or stronger than TX in the ORAC-FL method, while in the FRAP assay only four compounds (**1**–**4**) were found to have an antioxidant capacity better than AA. The results obtained by both methods differ significantly, however. In the case of the FRAP assay, the correlation of the antioxidant effect with AMPA affinity can be observed (Table 1). Compound **1**—the most potent AMPA antagonist within the entire series [22] and the most active phenylalanine in the FRAP test—as well as compound **8**—the most active one in the ORAC-FL test—were selected for further, cell-based neuroprotection studies. The data obtained showed the capability of **1** to protect SH-SY5Y and IMR-32 neuroblastoma cells from 6-OHDA-induced release of LDH from injured cells, whereas no neuroprotective effect of **8** was observed. Moreover, the phenylalanine **1** showed significant free-radical-scavenging properties compared to **8**, decreasing the level of ROS in SH-SY5Y cells after 3 h exposition on 500 µM of H_2_O_2_. Thus, the results of the performed cell-based assays seem to suggest that the FRAP method is more reliable in the search for antioxidants with desirable activity compared to the ORAC-FL test. Additionally, no toxic effects of **1** and **8** were observed within the concentration range 1–100 µM against both neuroblastoma cell lines. Furthermore, in the in vitro enzymatic assay, the MAO-B inhibition activity of compound **1** was determined (IC_50_ = 278 ± 29 nM), whereas no effect of compound **8** was found.

As shown in previous studies, AMPA receptors have been implicated in various physiological and pathophysiological phenomena, including learning and memory [55], mood disorders [8,56], pain [6,7] and seizures [13], and the therapeutic potential of AMPA receptor inhibition has been demonstrated by studies in which AMPA receptor antagonists have been shown to terminate seizures, relieve pain and attenuate mood disorders in animal models. In our present in vivo research, we focused on the pharmacological evaluation of the compound **1**, a novel AMPA receptor antagonist, to assess its effect on seizure and pain threshold in mice.

To determine the anticonvulsant potential of **1,** we used three distinct screening assays, i.e., MES, 6-Hz and PTZ tests. These tests are considered ‘gold standards’ in the search for novel AEDs as they allow predicting the efficacy of active anticonvulsant agents against different types of seizures in humans [57]. The MES test is useful for selection of drugs for generalized tonic-clonic seizures, whereas the 6-Hz psychomotor seizure model resembles psychomotor seizures occurring in human limbic epilepsy [57,58], and the PTZ model of clonic seizures generally refers to non-convulsive (*absence* or myoclonic) seizures in humans [57]. The results obtained in the present study demonstrated a distinct effect of the phenylalanine derivative **1** in these assays. The compound showed anticonvulsant properties in 6-Hz test; however, it was ineffective in the PTZ model or in MES test. This indicates that **1**, similar to levetiracetam, which is also active in this test [59], might have potential to attenuate psychomotor seizures without any effect on tonic-clonic (*grand mal*) seizures, *absence* or myoclonic seizures in humans.

This finding also excludes some additional mechanisms of action of the test compounds as agents active in the MES test that are thought to act via the blockade of voltage-gated sodium channels [57,58,60], whereas compounds showing activity in PTZ test might have a similar mechanism of action as ethosuximide—an antagonist of T-type voltage-gated calcium channels [57,58].

Oxidative stress has been implicated as a pathophysiological mechanism of drug-resistant epilepsy, and some oxidative stress parameters are closely linked with clinical variables [61]. Compounds with free-radical-scavenging properties have been shown to be effective in 6-Hz test in mice [62,63]. Such compounds might also attenuate chemotherapy-induced neuropathic pain [50,64,65,66]. In our study, the compound **1** showed antioxidant and anticonvulsant properties, but its analgesic properties in two mouse models of neuropathic pain were not demonstrated.

Taken together, the above data demonstrate compound **1** as an interesting phenylalanine-derived AMPA receptor antagonist with additional, desirable antioxidant, neuroprotective, MAO-B inhibitory properties and confirmed in vivo activity in selected anticonvulsant screening tests. It should be, however, noted that the experiments performed within this study have some limitations. Firstly, none of the in vitro models used here can fully mimic clinical features of PD or AD. Therefore, the neuroprotective and antioxidant potential of the test compound **1** should be interpreted only in terms of neurodegeneration rather than a specific disease. Secondly, as far as the performed in vivo assays are concerned, further extended studies must be planned to assess if the compound **1** confirms its neuroprotective properties in animal models of neurodegenerative diseases (e.g., 1-methyl-4-phenyl-1,2,3,6-tetrahydropyridine (MPTP) and 6-OHDA models of PD or rodent models of AD induced by scopolamine or MK-801).

## 4. Materials and Methods

The phenylalanine derivatives **1**–**22** have been synthesized as reported previously [20,21,22].

### 4.1. In Vitro Assays

#### 4.1.1. Antioxidant Activity

FRAP

The total antioxidant potential of aryl-substituted phenylalanine derivatives was determined using FRAP assay [34]. The FRAP reagent was prepared first by mixing acetate buffer (300 mM, pH 3.6), a solution of 10 mM TPTZ (2,4,6-tripyridyl-s-triazine, Sigma-Aldrich, Inc., St. Louis, MO, USA) in 40 mM HCl and 20 mM of iron (III) chloride hexahydrate (FeCl_3_ × 6H_2_O, Chempur, Piekary Śląskie, Poland) at 10:1:1 (*v*/*v*/*v*). The FRAP reagent (190 μL) and 1 mM of tested compound (10 μL) was added next to each well, mixed and incubated for 30 min. The absorbance was measured using a microplate reader EnSpire (PerkinElmer, Waltham, MA USA) at 593 nm. AA (1 mM, AMARA, Kraków, Poland) was used as the reference antioxidant. The results were expressed as % of ascorbic acid activity (%AAA). Analyses were performed in triplicate.

ORAC-FL

The ORAC-FL was performed according to procedure described in the literature [35]. The reagents used in this assay, TX, fluorescein and 2,2′-azobis(2-amidinopropane) dihydrochloride (AAPH), were purchased from Sigma-Aldrich, Inc., St. Louis, MO, USA. The prepared ORAC-FL reagent consisted of 0.3 μM of fluorescein and 100 µM of AAPH dissolved in PBS buffer. The compounds and the reference antioxidant TX were tested at the concentration 2.5 µM and the results were compared to the blank in which no antioxidant was present. The assays were performed in black 96-well plates. The fluorescence readings were taken by a microplate reader EnSpire (PerkinElmer, Waltham, MA, USA) every minute for a duration of 240 min. The excitation wavelength was 485 nm and the emission wavelength was 520 nm. The area under the fluorescence decay curve (AUC) was calculated next by GraphPad Prism™ software (version 5.01, San Diego, CA, USA). All measurements were performed in triplicate. The TX equivalent (TE) was calculated next using the following Equation (1):

TE = [(AUC_sample_ − AUC_blank_)/(AUC_TX_ − AUC_blank_)] × [C_TX_/C_sample_] (1)

#### 4.1.2. Neuroprotection

All cell culture protocols and assay conditions were applied as we reported previously [67]. SH-SY5Y (ATCC^®^ CRL-2266™) cell line was purchased from ATCC (Manassas, VA, USA). IMR-32 (ATCC^®^ CCL-127™) cell line was kindly provided by the Department of Oncogenomics, Academisch Medisch Centrum, Amsterdam, Holland. Before each test SH-SY5Y or IMR-32 cells were seeded in 96-well plates with transparent bottom at a concentration of 2.5 × 10^4^ cells/well in 100 μL of respective cell culture medium and cultured for 24 h to reach 70% confluence.

Cell Viability

The CellTiter 96^®^ AQueous Non-Radioactive Cell Proliferation Assay was purchased from Promega (Madison, WI, USA). After 24 h of cells incubation with compound **1** or **8** in the concentrations range 1–100 µM the MTS-based labeling mixture was added to each well and cells were incubated under the same conditions for 2–3 h. The absorbance was measured using a microplate reader EnSpire (PerkinElmer, Waltham, MA, USA) at 490 nm. All measurements were performed in triplicate and results are shown as mean ± SD.

Lactate Dehydrogenase Test

CytoTox-ONE™ Homogeneous Membrane Integrity Assay, which based on the LDH release, was purchased from Promega (Madison, WI, USA). SH-SY5Y or IMR-32 cells were seeded in 96-well white plates with transparent bottom. For neuroprotective studies, the cells were preincubated first for 1 h with compound **1**, **8** or reference antioxidants AA, TX (all at the concentration of 50 μM) and next the neurotoxin 6-OHDA (Sigma-Aldrich, Inc., St. Louis, MO, USA) was added at final concentration of 50 μM (SH-SY5Y assay) or 200 µM (IMR-32 assay). After 24 h of incubation, the CytoTox-ONE™ was added to each well and the cells were incubated next for 10 min at room temperature. The fluorescence was measured using a microplate reader EnSpire (PerkinElmer, Waltham, MA, USA) with an excitation wavelength of 560 nm and an emission wavelength of 590 nm. All measurements were performed in triplicate and results are shown as mean ± SD. The similar studies were also performed with SH-SY5Y cells injured by 100 μM of 6-OHDA (Figure S1, Supporting Materials).

ROS Assay

During this assay SH-SY5Y cells were preincubated first for 1 h with 250 μM solution of DCFH-DA (Sigma-Aldrich, Inc., St. Louis, MO, USA) in HBSS buffer. After DCFH-DA was removed, cells were washed with HBSS and incubated next for 1 h with 50 μM of compound **1** or **8**. Next, the H_2_O_2_ was added at final concentration 500 μM. After 3 h of incubation, the fluorescence was measured using a microplate reader EnSpire (PerkinElmer, Waltham, MA, USA) with an excitation wavelength of 486 nm and emission wavelength of 530 nm. All measurements were performed in triplicate and results are shown as mean ± SD.

#### 4.1.3. MAO-B Inhibition

Inhibitory potency of compound **1** and **8** on MAO-B isoenzyme was tested fluorometrically with a commercial Amplex™ Red Monoamine Oxidase Assay Kit (Thermo Fisher Scientific A12214, Waltham, MA, USA) as we reported previously [46,68]. Human MAO-B was purchased from Merck KGaA (Darmstadt, Germany). The activity of compound **1** and **8** was measured in the presence of p-tyramine (200 μM). In all experiments reference inhibitors in the concentrations that fully inhibited the MAO isoform were included: pargyline 10 μM and rasagiline 1 μM. Firstly, inhibitors’ activity was measured in a concentration of 1 μM. The results were normalized (no inhibition = 0% and fully inhibited enzyme 100%). For compounds that inhibited the enzyme by more than 50% further studies were conducted to obtain IC_50_ from concentration–response curves. All calculations were made in Microsoft Excel and GraphPadPrism software. All experiments were performed in duplicate and data are expressed as mean ± SEM.

### 4.2. In Vivo Assays

#### 4.2.1. Animals and Housing Conditions

Behavioral experiments were carried out in male Albino Swiss (CD-1) mice weighing 18–22 g. The mice were kept in cages, in groups of 10 animals. They had free access to food and water before experiments, and were maintained at room temperature 22–24 °C, under 12:12 (light/dark) cycle. For the assays the animals were selected randomly. After the experiments the animals were euthanized by cervical dislocation. Experimental procedures for in vivo tests were approved by the Local Ethics Committee of the Jagiellonian University in Krakow (Approval No. 33/2018; release date: 1.02.2018) and the treatment of animals was performed in full accordance with ethical standards laid down in respective Polish and EU regulations (Directive 2010/63/EU).

#### 4.2.2. Chemicals

Oxaliplatin was purchased from Activate Scientific GmbH (Prien, Germany). PTZ and STZ were provided by Merck KGaA (Darmstadt, Germany). For the in vivo tests, the compound **1** was suspended in 1% Tween 80 solution (Polskie Odczynniki Chemiczne, Gliwice, Poland), then being administered intraperitoneally 1 h before tests. For the evaluation of anticonvulsant properties of the test compound, a fixed dose of 100 mg/kg, was used. Since many AEDs at lower doses are used as analgesic drugs, for pain tests the doses 10 and 30 mg/kg were selected.

#### 4.2.3. Assessment of Anticonvulsant Activity

Maximal Electroshock Test (MES Test)

MES test was performed as described previously [69]. In this test mice received an electric stimulus (25 mA, 0.2 s, 50 Hz) via auricular electrodes connected to an electroshock generator (Hugo Sachs rodent shocker, Hugo Sachs Elektronik, Germany). A tonic extension of hind limbs was the endpoint in this assay.

PTZ Test

This test was carried out according to the method recently described [69]. Clonic seizures were induced by a subcutaneous injection of PTZ (Merck KGaA, Darmstadt, Germany) used at the dose of 100 mg/kg. After the injection of PTZ the animals were put separately into glass beakers and were observed for the next 30 min. In this assay the latency to the occurrence of the first clonic seizure episode was measured and compared in drug-treated and control mice. Clonic seizures were defined as a clonus of the whole body that lasted more than 3 s, and was accompanied by the loss of the righting reflex. The number of seizure episodes was also recorded in each group.

6-Hz Test

This test was performed according to Barton et al. [70]. It is an alternative electroshock paradigm that involves low-frequency (6 Hz), long-duration (3 s) electrical stimulation. Corneal stimulation (0.2 ms-duration monopolar rectangular pulses at 6 Hz for 3 s) was delivered by a constant-current device (Ugo Basile, Italy). During electrical stimulation mice were manually restrained and released into the observation cage immediately after current application. At the time of drug administration, a drop of 0.5% tetracaine (Altacaine sterile solution, Altaire Pharmaceuticals Inc., Aquebogue, NY, USA) was applied into animal eyes. Prior to the placement of corneal electrodes, a drop of 0.9% saline was applied on the eyes. In this model seizures manifest in ‘stunned’ posture associated with rearing, forelimb automatic movements and clonus, twitching of the vibrissae and Straub tail. At the end of the seizure episode the animals resume their normal exploratory behavior. In this test protection against a seizure episode is considered the end point and animals are considered to be protected if they resume their normal exploratory behavior within 10 s after electrical stimulation [71].

#### 4.2.4. Assessment of Antiallodynic and Antihyperalgesic Properties in Neuropathic Pain Models

##### Streptozotocin-Induced Painful Diabetic Neuropathy

Induction of Diabetes

To induce type I diabetes and diabetic neuropathic pain, mice were administered STZ, an alkylating antitumor drug that destroys insulin-secreting islet cells. Mice were administered a single injection of STZ (200 mg/kg, i.p.). Age-matched control mice received an equal volume of citrate buffer. Blood glucose levels were measured 1 day before (referred to as ‘day 0′) and repeatedly 1, 2 and 3 weeks after STZ injection using a blood glucose monitoring system (AccuChek Active, Roche, France). Blood samples (5 µL) for the measurement of glucose concentration were obtained from the tail vein of the mice. The animals were considered to be diabetic when their blood glucose concentration exceeded 300 mg/dl. Pain tests in diabetic mice were performed 3 weeks after STZ administration [49].

Influence on Tactile Allodynia in Diabetic Mice—Von Frey Test

The electronic von Frey unit (Bioseb, France) is supplied with a single flexible filament applying increasing force (from 0 to 10 g) against the plantar surface of the hind paw. In this assay the nocifensive paw withdrawal response automatically turns off the stimulus and the mechanical pressure that evoked the response was recorded. On the day of the experiment, diabetic mice were placed individually in test compartments with a wire mesh bottom and were allowed to habituate for 1 h. After the habituation period, in order to obtain baseline values, each mouse was tested 3 times alternately in each hind paw, allowing at least 30 s between each measurement. Then, the mice were intraperitoneally pretreated with test compounds or vehicle and 60 min later the animals were tested again and 3 measures were taken and averaged to obtain mean post drug values for each mouse [49,72].

Influence on Heat Hyperalgesia in Diabetic Mice—Hot Plate Test

The effect of **1** on heat hyperalgesia was assessed using the hot plate test. In this assay, after the establishment of predrug latency to pain reaction for each animal, diabetic mice were treated with the test compounds. Sixty minutes later, they were placed on the hot plate apparatus (Hot/cold plate, Bioseb, France) set at 55–56 °C and were observed to the appearance of a nocifensive response (hind paw licking or jumping). Cut-off of 60 s was established to avoid paw tissue damage, and mice not responding within 60 s were removed from the apparatus and assigned a score of 60 s [72].

##### Oxaliplatin-Induced Peripheral Neuropathy

Induction of Neuropathy

In the chemotherapy-induced peripheral neuropathy model, in order to induce neuropathic pain, oxaliplatin was administered i.p. to mice as a single dose of 10 mg/kg. Pain tests were performed 3 h and 7 days later to assess whether the test compounds affect early phase and late-phase tactile allodynia and cold hyperalgesia.

Influence on Tactile Allodynia in Oxaliplatin-Treated Mice—Von Frey Test

This test was performed according to a method used to study the effect of compound **1** on tactile allodynia in the mouse diabetic neuropathic pain model.

Influence on Cold Hyperalgesia in Oxaliplatin-Treated Mice—Cold Plate Test

The effect of **1** on cold pain threshold was assessed using the cold plate test [73,74]. In this assay, after the establishment of predrug latency to pain reaction for each animal, the mice were treated with the test compounds and 60 min later they were placed on the cold plate apparatus (Hot/cold plate, Bioseb, France) set at 2.5 °C and were observed for the appearance of a nocifensive response (hind paw licking, shaking, jumping or abnormal movements). Cut-off of 60 s was established to avoid paw tissue damage, and mice not responding within 60 s were removed from the apparatus and assigned a score of 60 s.

#### 4.2.5. Statistical Analysis

Data analysis of in vivo results was carried out using GraphPad Prism software (version 9.0, San Diego, CA, USA). Numerical results are expressed as the mean ± SEM. Statistical analysis was carried out by Student’s *t*-test or repeated measures analysis of variance (ANOVA), followed by Tukey’s post hoc comparison.

## Figures and Tables

**Table 2:** Anticonvulsant activity of the compound **1** in MES test.

Compound	Dose [mg/kg]	X/Y ^1^	Mortality Rate (%)
Vehicle	-	1/5	20
1	100	0/4	25

^1^ Results are shown as the number of mice protected (X) per the number of mice tested (Y).

**Table 3:** Anticonvulsant activity of the compound **1** in 6-Hz test.

Compound	Dose [mg/kg]	X/Y ^1^	Mortality rate (%)
Vehicle	-	0/3	0
1	100	3/4	0

^1^ Results are shown as number of mice protected (X) per number of mice tested (Y).

## Data Availability

Data is contained within the article or supplementary material to this article.

## Supplementary Materials

The following supporting information can be downloaded. Figure S1: Neuroprotection studies of compounds **1** and **8** (50 µM) against 6-OHDA (100 µM) - induced LDH release from necrotic SH-SY5Y cells. Ascorbic acid (AA, 50 µM) and Trolox (TX, 50 µM) were used as the reference antioxidants. Statistical significance was evaluated by one-way ANOVA, followed by Bonferroni’s comparison (ns—not significant, compared with 100 µM of 6-OHDA); Table S1: Selected physicochemical descriptors calculated by SwissADME calculator for individual enantiomers of compounds **1**, **8** as well as levodopa ((*S*)-3,4-dihydroxyphenylalanine); Table S2. Selected pharmacokintetic descriptors calculated by SwissADME calculator for individual enantiomers of compounds **1**, **8** as well as levodopa.

## Sample Availability

Samples of the compounds **1**–**22** are available from the authors.
