# Supplementary material for: Phenylalanine-Based AMPA Receptor Antagonist as the Anticonvulsant Agent with Neuroprotective Activity—In Vitro and In Vivo Studies
Source: Molecules. 2022 Jan 27;27(3):875. doi: 10.3390/molecules27030875 (PMC8840081; doi:10.3390/molecules27030875)
Supplement: Supplementary file 1 [file molecules-27-00875-s001.zip › molecules-1535907-supplementary-done.pdf]

molecules

Supporting Materials

Phenylalanine-based AMPA receptor antagonist as the anticonvulsant agent with neuroprotective activity - *in vitro* and *in vivo* studies

Gniewomir Latacz [1], Kinga Sałat [2] , Anna Furgała-Wojas [2], Adrian Martyniak [1], Agnieszka Olejarz-Maciej [1], Ewelina Honkisz-Orzechowska [1] and Ewa Szymańska [1,*]

[1] Department of Technology and Biotechnology of Drugs, Jagiellonian University Medical College, 9 Medyczna St., 30-688 Krakow, Poland
[2] Department of Pharmacodynamics, Faculty of Pharmacy, Jagiellonian University Medical College, 9 Medyczna St., 30-688 Krakow, Poland
* Correspondence: ewa.szymanska@uj.edu.pl

Content:

Neuroprotection studies in SH-SY5Y against 100 μM 6-OHDA……………………………………………2

Selected physicochemical and pharmacokinetic parameters predicted in silico for **1** and **8**…………3

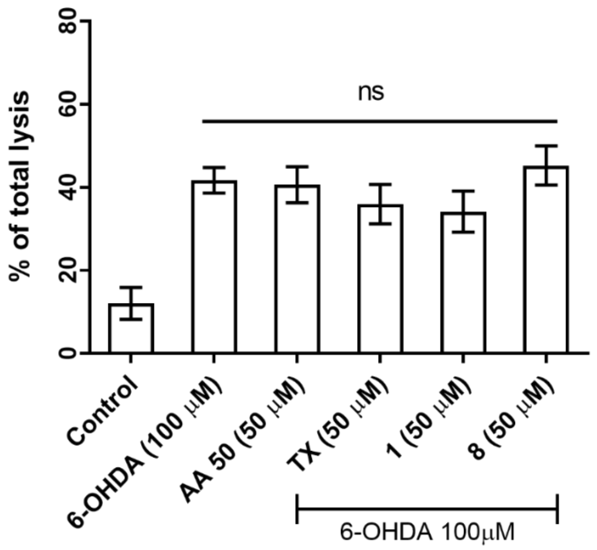

Figure S1. Neuroprotection studies of compounds **1** and **8** (50 μM) against 6-OHDA (100 μM) - induced LDH release from necrotic SH-SY5Y cells. Ascorbic acid (AA, 50 μM) and Trolox (TX, 50 μM) were used as the reference antioxidants. Statistical significance was evaluated by one-way ANOVA, followed by Bonferroni's comparison (ns – not significant, compared with 100 μM of 6-OHDA).

Table S1. Selected physicochemical descriptors calculated by SwissADME calculator for individual enantiomers of compounds **1**, **8** as well as **levodopa** ((*S*)-3,4-dihydroxyphenylalanine)

Symbol	Molecular weight [g/mol]	Lipophilicity LogP*	Solubility LogS**	TPSA*** [Å²]
(*S*)-**1**	352.73	0.47	-2.65 (soluble)	149.60
(*R*)-**1**	352.73	0.50	-2.65 (soluble)	149.60
(*S*)-**8**	306.74	1.07	-1.66 (very soluble)	109.57
(*R*)-**8**	306.74	1.06	-1.66 (very soluble)	109.57
Levodopa	197.19	-0.86	1.11 (highly soluble)	103.78

*Average of five methods implemented in SwissADME website; **Topological method implemented from Ali J. et al, 2012, *J Chem Inf Model*, *** Topological Polar Surface Area, calculated from Ertl P. et al, 2000, *J Med Chem*.

Table S2. Selected pharmacokintetic descriptors calculated by SwissADME calculator for individual enantiomers of compounds **1**, **8** as well as **levodopa**

Symbol	GI absorption	BBB permeant	P-gp substrate	Inhibitor				
				CYP1A2	CYP2C19	CYP2C9	CYP2D6	CYP3A4
(*S*)-**1**	low	no	no	no	no	no	no	no
(*R*)-**1**	low	no	no	no	no	no	no	no
(*S*)-**8**	high	no	no	no	no	no	no	no
(*R*)-**8**	high	no	no	no	no	no	no	no
Levodopa	high	no	no	no	no	no	no	no
